# ChatGPT's performance in sample size estimation: a preliminary study on the capabilities of artificial intelligence

**DOI:** 10.1093/fampra/cmaf069

**Published:** 2025-08-26

**Authors:** Paul Sebo, Ting Wang

**Affiliations:** University Institute for Primary Care (IuMFE), University of Geneva, 1211 Geneva, Switzerland; School of Library and Information Management, Emporia State University, Emporia, KS 66801, United States

**Keywords:** accuracy, artificial intelligence, ChatGPT, performance, reproducibility, sample size

## Abstract

**Background:**

Artificial intelligence tools, including large language models such as ChatGPT, are increasingly integrated into clinical and primary care research. However, their ability to assist with specialized statistical tasks, such as sample size estimation, remains largely unexplored.

**Methods:**

We evaluated the accuracy and reproducibility of ChatGPT-4.0 and ChatGPT-4o in estimating sample sizes across 24 standard statistical scenarios. Examples were selected from a statistical textbook and an educational website, covering basic methods such as estimating means, proportions, and correlations. Each example was tested twice per model. Models were accessed through the ChatGPT web interface, with a new independent chat session initiated for each round. Accuracy was assessed using mean and median absolute percentage error compared with validated reference values. Reproducibility was assessed using symmetric mean and median absolute percentage error between rounds. Comparisons were performed using Wilcoxon signed-rank tests.

**Results:**

For ChatGPT-4.0 and ChatGPT-4o, absolute percentage errors ranged from 0% to 15.2% (except one case: 26.3%) and 0% to 14.3%, respectively, with most examples showing errors below 5%. ChatGPT-4o showed better accuracy than ChatGPT-4.0 (mean absolute percentage error: 3.1% vs. 4.1% in round#1, *P*-value = .01; 2.8% vs. 5.1% in round#2, *P*-value =.02) and lower symmetric mean absolute percentage error (0.8% vs. 2.5%), though not significant (*P*-value = .18).

**Conclusions:**

ChatGPT-4.0 and ChatGPT-4o provided reasonably accurate sample size estimates across standard scenarios, with good reproducibility. However, inconsistencies were observed, underscoring the need for cautious interpretation and expert validation. Further research should assess performance in more complex contexts and across a broader range of AI models.

Key messages

- This study assessed ChatGPT's accuracy and consistency in sample size estimation
- Most errors were under 5%, some exceeded 10%
- ChatGPT-4o showed lower error rates than ChatGPT-4.0
- Estimates were generally consistent between rounds
- Expert review is needed before relying on outputs

## Introduction

Artificial intelligence (AI) plays an increasingly prominent role in healthcare and biomedical research, offering novel tools for data analysis, knowledge generation, and decision support across diverse clinical and research domains [1–3]. Large language models (LLMs) have further expanded these possibilities, particularly in clinical decision support, healthcare administration, and medical education, through advanced natural language processing and text generation [4–9]. In particular, ChatGPT—an interactive LLM developed by OpenAI, trained on massive amounts of text data using the Generative Pre-trained Transformer (GPT) architecture—has attracted growing attention for its ability to generate coherent, human-like text, answer questions, and engage in natural language conversations, with potential applications in these domains [10–17].

While these general capabilities have made ChatGPT and other LLMs widely accessible, their potential to assist with more structured, technical tasks—particularly those involving statistical reasoning—remains underexplored. Recent studies have begun investigating the use of ChatGPT in research workflows, including support for systematic reviews, hypothesis generation, drafting of protocols and manuscripts, and basic statistical analysis [18–22]. These exploratory efforts suggest that ChatGPT may not only be helpful for streamlining some stages of research design but also highlight challenges related to accuracy, transparency, and reproducibility.

One such technical task is sample size estimation, a fundamental step in research planning that directly impacts statistical power, study validity, and ethical use of resources [23]. Unlike tasks such as summarization or paraphrasing, sample size calculations require precise numerical reasoning, correct interpretation of statistical parameters, and application of appropriate formulas. These features make sample size estimation a particularly relevant and rigorous test case for evaluating the capabilities of ChatGPT in quantitative domains.

To date, few studies have evaluated ChatGPT's performance in generating valid sample size estimates [24, 25]. Existing analyses have typically been limited in scope, focusing on a small number of scenarios or illustrative examples. However, as ChatGPT become increasingly embedded in research workflows—including in primary care and low-resource settings where access to statistical expertise may be limited—it is critical to assess whether the tool can be relied upon for core methodological tasks.

Methnani *et al*.[24] evaluated ChatGPT's performance on four sport-science and sport-medicine studies requiring sample size calculations (three randomized controlled trials and one survey study). ChatGPT-3.5 correctly calculated the sample size in one randomized trial but failed in the other three cases. In one survey example, it applied the wrong formula; only after further interaction was the correct sample size obtained. Additionally, when one prompt was repeated, ChatGPT-3.5 generated a different result than in its initial response, highlighting a lack of reproducibility. In another study, Ignjatović and Stevanović [25] assessed ChatGPT's performance on 10 biostatistics questions—including one on sample size estimation—in a medical education context. On the initial attempt, GPT-3.5 correctly answered five questions and GPT-4 answered six. After up to 3 iterative prompts, GPT-3.5 improved to 7 correct answers, while GPT-4 ultimately solved all 10 questions correctly. For the sample size estimation problem, GPT-3.5 failed to provide a correct calculation even after three rounds, whereas GPT-4 reached the correct answer only after the third round.

Building on this early work, the present study aimed to evaluate the accuracy and reproducibility of ChatGPT-4.0 and ChatGPT-4o in estimating sample sizes across 24 standard statistical scenarios. By comparing AI-generated results with validated reference values, we seek to clarify the strengths and limitations of generative AI in supporting core elements of research methodology, and to identify areas where expert oversight remains essential.

## Methods

### Data sources and rationale

We used two publicly available resources to construct a standardized set of sample size estimation problems: (i) a statistical textbook by Verma and Verma [26] and (ii) the Sample Size Calculator website by Arifin [27]. These sources were chosen because they provide a wide variety of clearly documented sample size examples involving commonly used statistical tests. The examples in Verma and Verma's book were primarily computed using G*Power, while Arifin's website examples were predominantly generated using the Sample Size Calculator. Peer-reviewed articles were not used, as they often omit full parameter specifications required to verify or reproduce sample size calculations, making them unsuitable for systematic evaluation.

### Sampling strategy

From Verma and Verma's textbook, we limited selection to the first six chapters, which cover foundational topics such as estimating means, proportions, differences between groups, correlations, and simple regression. To define the number of examples to extract, we chose a range between 10 and 20: fewer than 10 would not provide enough variety, while more than 20 examples would risk redundancy or saturation. Rather than selecting a fixed number ourselves—thus reducing the potential for unconscious bias (e.g. ending selection when ChatGPT performance appears to worsen)—we generated a random number in that range, which resulted in a total of 14 examples. These were then randomly selected from the eligible chapters and labeled V1–V14.

In addition, we included the first 10 examples from Arifin's website, labeled A1–A10, covering similarly standard use cases. All examples focused exclusively on basic statistical problems that do not require expert tools or advanced methods, representing typical challenges faced by researchers working without statistical support. This process yielded 24 unique examples for testing (see Table 1 and Supplementary Material for full descriptions).

**Table 1.** The 24 examples included in the study to test the accuracy of ChatGPT in estimating sample size requirements, categorized by statistical analysis and sample size method.

Example number^a^	Title of the exercise	Statistical analysis	Sample size method
V1	Estimating mean height of male students	Mean, 95% confidence interval	Single mean—estimation
V2	Estimating mean serum bilirubin levels in infants	Mean, 95% confidence interval	Single mean—estimation
V3	Estimating the proportion of non-vegetarians	Proportion, 95% confidence interval	Single proportion—Estimation
V4	Estimating the proportion of non-smokers	Proportion, 95% confidence interval	Single proportion—estimation
V5	Testing VO₂ max hypothesis	Independent *t*-test	Single mean—hypothesis testing
V6	Testing mean WBC count differences	Independent *t*-test	Two means—hypothesis testing
V7	Detecting calcium concentration differences	Independent *t*-test	Single mean—hypothesis testing
V8	Comparing noise levels in two settings	Independent *t*-test	Two means—hypothesis testing
V9	Evaluating weight loss program effectiveness	Paired *t*-test	Two means (paired)—hypothesis testing
V10	Comparing study hours using Mann–Whitney test	Mann–Whitney test	Two means (non-parametric)—Hypothesis testing
V11	Comparing vaccination proportions	Chi-squared test	Two proportions—hypothesis testing
V12	Testing correlation between respiratory rate and Fat%	Pearson's correlation coefficient	Single Pearson's correlation—hypothesis testing
V13	Testing height-weight correlation in children	Pearson's correlation coefficient	Single Pearson's correlation—hypothesis testing
V14	Comparing correlations in men and women	Pearson's correlation coefficient	Two Pearson's correlations—hypothesis testing
A1	Estimating mean BMI among students	Mean, 95% confidence interval	Single mean—estimation
A2	Estimating mean height of male students estimating prevalence of obesity among students	Proportion, 95% confidence interval	Single proportion—estimation
A3	Comparing mean BMI between students	Independent *t*-test	Two means—hypothesis testing
A4	Comparing prevalence of obesity between students from different years	Chi-squared test	Two proportions—hypothesis testing
A5	Comparing mean weight before and after a weight loss program	Paired *t*-test	Two means (paired)—Hypothesis testing
A6	Comparing percentage of vaccine uptake before and after a vaccine awareness campaign	McNemar's test	Two proportions (paired)—Hypothesis testing
A7	Determining Pearson's correlation between age and cholesterol level	Pearson's correlation coefficient	Single Pearson's correlation—hypothesis testing
A8	Identifying factors associated with cholesterol levels	Multiple linear regression	Rule-of-thumb—10 subjects per independent variable
A9	Identifying factors associated with hypertension in employees	Multiple logistic regression	Rule-of-thumb—10 events per parameter
A10	Exploring the Internal structure validity of ABC-Q	Exploratory factor analysis	Rule-of-thumb—5 respondents per item

^a^Examples V1 to V14 are sourced from Verma's book, selected at random from the first six chapters. Examples A1 to A10 are sourced from Arifin's website, specifically the first 10 examples listed. All examples were chosen to focus on cases that do not require advanced statistical methods or specialized sample size estimation tools.

### Prompt structure and testing procedure

Each example was reworded to reduce the likelihood that it had been encountered in ChatGPT's training data. All reference sample sizes were independently verified by a professional statistician (T. W.). For each example, T. W. reviewed the solution provided in the source (Verma's book or Arifin's website), confirmed that the proposed test and formula were appropriate for the scenario, and repeated the calculations using the given parameters. In all cases, T. W. obtained results identical to those reported in the sources, thereby confirming the accuracy of the reference values used for this study.

ChatGPT-4.0 and ChatGPT-4o were accessed through https://chat.openai.com/. For each model, estimations were conducted in two independent rounds, separated by a 1-day interval: on 25 and 27 January, 2025 for GPT-4.0, and 25 and 27 April, 2025 for GPT-4o. A fresh chat session was started for each prompt to minimize memory effects. The prompt format was standardized as:

“*Please calculate the required sample size for the following scenario: [full example text]*”.

No further clarification or statistical test was provided; the prompt contained all information needed to infer the correct formula.

### Evaluation metrics and statistical analysis

To evaluate accuracy, defined as the closeness of ChatGPT's sample size estimates to the validated reference values, we computed the percentage error by dividing the difference between the estimated and true sample sizes by the true value and multiplying by 100. To assess reproducibility, defined as the consistency of ChatGPT's estimates across two independent testing rounds, we calculated the symmetric percentage error by dividing the difference between the second-round and first-round estimates by their mean and multiplying by 100. For examples reporting sample size per group, total sample size was computed by summing the groups. Percentage errors were then calculated using these total values to ensure consistency across examples, including those with unequal group sizes.

To explore the directionality of estimation errors, we examined the number of positive and negative values among the signed percentage errors, to assess whether the models tended to systematically overestimate or underestimate sample sizes.

We reported both mean and median values for each metric after removing the sign of each individual percentage error or symmetric percentage error: mean absolute percentage error (MAPE) and median absolute percentage error (MdAPE) for accuracy, and symmetric MAPE (sMAPE) and symmetric MdAPE (sMdAPE) for reproducibility. Standard deviations (SDs) and interquartile ranges (IQRs) were reported to describe variability. Given the small sample size and non-normality of the data, the Wilcoxon signed-rank test was used to compare MdAPE values between ChatGPT-4.0 and ChatGPT-4o separately for Rounds 1 and 2, and to compare sMdAPE values between the two models.

To further quantify differences in accuracy between models, we calculated the paired differences in absolute percentage errors for each example between ChatGPT-4.0 and ChatGPT-4o. These paired differences were summarized using the mean (SD) and median (IQR) to describe effect sizes.

A *P*-value <.05 was considered statistically significant. All statistical analyses were performed using Stata version 15.1.

### Ethical considerations

All data used in this study consist of publicly available sample size examples from Verma's textbook and Arifin's website. No individual-level, personal, or sensitive data were used. During data collection, only the final sample size values generated by ChatGPT were recorded locally; full ChatGPT outputs (including explanations) were not stored. This study did not involve the collection or use of any personal or health-related data; therefore, no ethical approval was required under Swiss law. In accordance with the FAIR principles (Findable, Accessible, Interoperable, and Reusable), all relevant data are openly shared and fully documented to facilitate transparency, reproducibility, and reuse by other researchers.

## Results

**Figure 1.**
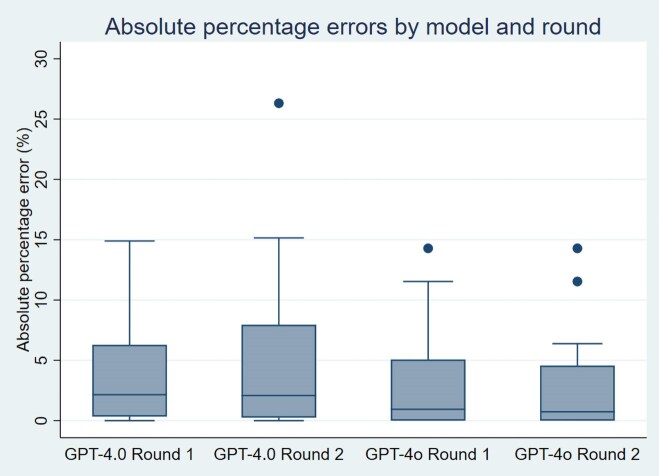
Boxplot of absolute percentage errors for ChatGPT-4.0 and ChatGPT-4o across two rounds of sample size estimation.

The main results are presented in Table 2. Figure 1 displays boxplots of absolute percentage errors, providing a visual comparison of model accuracy across both rounds. For ChatGPT-4.0, absolute percentage errors ranged from 0% to 15.2%, except for example V12 in round two (26.3%). Among the 24 examples, 4 showed no error in the first round and 5 in the second; 16 and 17 examples, respectively, had a difference below 5%, while 4 and 6 examples exceeded 10%. For ChatGPT-4o, absolute percentage errors ranged from 0% to 14.3%. Ten examples showed no error in the first round and 9 in the second; 18 and 19 examples, respectively, had a difference below 5%, and 3 and 2 examples exceeded 10%.

**Table 2.** Comparison of the accuracy and reproducibility of ChatGPT-4.0 and ChatGPT-4o in estimating sample sizes across 24 examples.

Example number^a^	Actual sample size^b^	ChatGPT-4.0 estimated sample size (first round)	Percentage error (first round)^c^	ChatGPT-4.0 estimated sample size (second round)	Percentage error (second round)^c^	Symmetric percentage difference between rounds (ChatGPT-4.0)^d^	ChatGPT-4o estimated sample size (first round)	Percentage error (first round)^c^	ChatGPT-4o estimated sample size (second round)	Percentage error (second round)^c^	Symmetric percentage difference between rounds (ChatGPT-4o)^d^
V1	24	25	4.2	25	4.2	0	25	4.2	25	4.2	0
V2	26	23	−11.5	23	−11.5	0	23	−11.5	23	−11.5	0
V3	504	505	0.2	505	0.2	0	505	0.2	505	0.2	0
V4	897	897	0	897	0	0	897	0	897	0	0
V5	19	18	−5.3	18	−5.3	0	18	−5.3	18	−5.3	0
V6	24/group	23/group	−4.2	23/group	−4.2	0	23/group	−4.2	23/group	−4.2	0
V7	41	38	−7.3	39	−4.9	2.6	39	−4.9	39	−4.9	0
V8	42/group	41/group	−2.4	41/group	−2.4	0	41/group	−2.4	42/group	0	2.4
V9	21	22	4.8	24	14.3	8.7	24	14.3	24	14.3	0
V10	47/group	42/group	−10.6	42/group	−10.6	0	42/group	−10.6	49/group	4.3	15.4
V11	636 and 795	603 and 754	−5.2	622 and 777	−2.2	3.0	622 and 778	−2.2	621 and 776	−2.4	−0.2
V12	19	17	−10.5	24	26.3	34.1	19	0	19	0	0
V13	129	131	1.6	127	−1.6	−3.1	129	0	130	0.8	0.8
V14	251/group	248/group	−1.2	251/group	0	1.2	248/group	−1.2	248/group	−1.2	0
A1	272	271	−0.4	271	−0.4	0	272	0	272	0	0
A2	322	321	−0.3	321	−0.3	0	322	0	321	−0.3	−0.3
A3	52/group	51/group	−1.9	51/group	−1.9	0	52/group	0	52/group	0	0
A4	189/group	186/group	−1.6	186/group	−1.6	0	189/group	0	189/group	0	0
A5	23	23	0	23	0	0	23	0	23	0	0
A6	47	40	−14.9	40	−14.9	0	44	−6.4	44	−6.4	0
A7	33	30	−9.1	28	−15.2	−6.9	35	6.1	35	6.1	0
A8	88	88	0	88	0	0	88	0	88	0	0
A9	382	382	0	382	0	0	382	0	382	0	0
A10	288	286	−0.7	286	−0.7	0	286	−0.7	286	−0.7	0
MAPE (SD)^e^			4.1 (4.4)		5.1 (6.8)			3.1 (4.2)		2.8 (3.9)	
sMAPE (SD)^f^						2.5 (7.1)					0.8 (3.2)
MdAPE (IQR)^g^			2.2 (0.3–6.3)		2.1 (0.3–8.0)			1.0 (0–5.1)		0.7 (0–4.6)	
sMdAPE (IQR)^h^						0 (0–1.9)					0 (0–0)

^a^Examples V1 to V14 are sourced from Verma's book, selected at random from the first six chapters. Examples A1 to A10 are sourced from Arifin's website, specifically the first ten examples listed. All examples were chosen to focus on cases that do not require advanced statistical methods or specialized sample size estimation tools.

^b^Samples sizes were verified by a professional statistician.

^c^Percentage error (for accuracy) is computed by dividing the difference between the estimated and true sample sizes by the true sample size, and multiplying by 100.

^d^Symmetric percentage difference (for reproducibility) is computed by dividing the difference between the second-round and first-round estimates by their mean, and multiplying by 100.

^e^MAPE, mean absolute percentage error (for accuracy); it is the mean of all percentage errors across all examples, after removing the sign.

^f^sMAPE, symmetric mean absolute percentage error (for reproducibility); it is the mean of all symmetric percentage differences across all examples, after removing the sign.

^g^MdAPE, median absolute percentage error (for accuracy); it is the median of all percentage errors across all examples, after removing the sign. Wilcoxon signed-rank test: *P* = .01 for the first-round comparison; *P* = .02 for the second-round comparison.

^h^sMdAPE, symmetric median absolute percentage error (for reproducibility); it is the median of all symmetric percentage differences across all examples, after removing the sign. Wilcoxon signed-rank test: *P* = .18 for the comparison between ChatGPT-4.0 and ChatGPT-4o.

Reproducibility between rounds showed variation ranging from 0% to 8.7% for ChatGPT-4.0 (except V12: 34.1%), and from 0% to 2.4% for ChatGPT-4o (except V10: 15.4%). No variation was observed in 17 examples for ChatGPT-4.0 and 19 for ChatGPT-4o.

In terms of error direction, ChatGPT-4.0 tended to underestimate sample sizes, with 16 negative, 4 positive, and 4 zero-error cases in Round 1, and 15 negative, 4 positive, and 5 zero-error cases in Round 2. ChatGPT-4o showed a more balanced distribution, although negative values still outnumbered positive ones: 10 negative, 4 positive, and 10 zero-error cases in Round 1; 9 negative, 6 positive, and 9 zero-error cases in Round 2.

Overall, ChatGPT-4o demonstrated significantly better accuracy than ChatGPT-4.0, with lower MAPE values in both rounds (first round: 3.1% vs. 4.1%, *P*-value = .01; second round: 2.8% vs. 5.1%, *P*-value = .02). Although ChatGPT-4o also showed lower variability between rounds in terms of sMAPE (0.8% vs. 2.5%), this difference was not statistically significant (*P*-value = .18). ChatGPT-4o further exhibited lower MdAPE and a narrower IQR, indicating greater stability and closer agreement with reference values. The sMdAPE of 0% (IQR: 0–0) indicates identical outputs across rounds for most examples, reflecting high reproducibility.

Paired differences in absolute percentage errors between ChatGPT-4.0 and ChatGPT-4o showed a mean difference of 1.0% (SD 3.5) in Round 1 and 2.3% (SD 5.8) in Round 2, favoring ChatGPT-4o. Median differences were 0% (IQR: 0%–1.8%) across both rounds, indicating identical estimates in the median case. However, Wilcoxon signed-rank tests revealed statistically significant shifts favoring ChatGPT-4o (*P*-values = .01 in Round 1 and .02 in Round 2). Specifically, ChatGPT-4o outperformed ChatGPT-4.0 in 10 and 9 examples in Rounds 1 and 2, respectively, while ChatGPT-4.0 was superior in only 1 and 2 examples.

## Discussion

### Summary of key findings

This study evaluated the accuracy and reproducibility of ChatGPT-4.0 and ChatGPT-4o in estimating sample sizes across various statistical scenarios. Percentage differences between AI-generated and reference values ranged from 0% to 15.2% (except one case: 26.3%) for ChatGPT-4.0 and 0% to 14.3% for ChatGPT-4o, with most examples showing differences below 5%. While reproducibility between rounds was high overall, some inconsistencies were noted. ChatGPT-4o demonstrated slightly better accuracy and reproducibility than ChatGPT-4.0, although the difference in reproducibility was not statistically significant. Both models tended to underestimate sample sizes, particularly ChatGPT-4.0.

### Comparison with existing literature

Limited data are available in the literature for comparison, with one study evaluating four sport-science and sport-medicine examples [24] and another assessing a single sample size estimation in a medical education setting [25], both showing mixed results.

The differences observed in our study may not necessarily reflect errors by ChatGPT but could arise from multiple contributing factors. These include variations in statistical approaches (e.g. test selection, assumptions, thresholds, calculation parameters); occasional simplification or misapplication of formulas; reliance on heuristics rather than exact computation; a tendency to generate conservative or “safe” estimates when uncertain; and training on textual data where larger effect sizes are more commonly reported. Unlike dedicated statistical software, LLMs do not perform formal verification steps and may omit design complexities that typically influence sample size calculations. Since ChatGPT was provided with complete and correct input data in all examples, these limitations likely reflect how LLMs internally process and apply statistical reasoning. Several of these mechanisms—particularly simplified formulas, heuristic reasoning, conservative bias, and optimism in effect size assumptions—may specifically contribute to the observed underestimation of required sample sizes.

Our study did not investigate the relative contribution of these factors in depth. Future research should explore these mechanisms to better understand how ChatGPT interprets problem structures, applies statistical reasoning, and handles contextual information during sample size estimation.

### Practical implications and future directions

In primary care and clinical research settings, where statistical resources may be limited, AI-based tools could offer a rapid preliminary check. Agreement between AI-generated estimates and the researcher's calculations may provide reassurance, while discrepancies should prompt caution and expert review. However, our findings emphasize that ChatGPT should not be used as a standalone statistical tool. Unlike established software such as G*Power, ChatGPT lacks transparency in its methodology, does not always apply standard approaches, and may miss critical statistical nuances. These limitations are consistent with the current literature stressing the need for human oversight when using AI for complex analytical tasks [28]. In addition, institutions should develop clear usage guidelines to integrate ChatGPT and other LLMs into research practice responsibly, including recommendations for validating AI-generated estimates with established methods, transparently disclosing AI involvement in study planning, and providing training for researchers to understand both the capabilities and constraints of such models.

Future research should evaluate the performance of newer AI models optimized for numerical reasoning and conduct direct comparisons across different generative AI tools. Larger and more diverse sets of sample size problems, including complex study designs, should be tested. In particular, larger datasets would enable subgroup analyses according to test type (e.g. *t*-tests, chi-squared tests, correlation, regression) and problem complexity, providing a more detailed understanding of areas where model performance varies and helping to better characterize strengths and weaknesses. Investigating how AI-generated sample size estimations could be integrated into real-world research workflows—as preliminary aids or verification steps—would also be valuable.

In addition, future work should include a qualitative, case-by-case analysis of the explanations provided by ChatGPT, regardless of whether the final answer is correct or incorrect. Such investigation is essential to better understand how the model reasons, selects statistical tests, applies formulas, and handles approximations. For incorrect answers, contributing mechanisms may include misunderstanding the question, choosing an inappropriate test, misapplying a correct method, or oversimplifying calculations. For correct answers, similar pitfalls may still exist; the model may occasionally arrive at the correct number for incorrect or coincidental reasons. This type of detailed interpretative work is highly time-consuming and would benefit from collaboration between statisticians and qualitative researchers.

### Limitations

This study has some limitations. First, it included only examples that did not require advanced statistical methods or professional software, which may limit the generalizability of the findings to more complex calculations, such as those involving survival analysis, mixed models, or advanced non-parametric tests, and the small sample size of 24 examples may not capture the full variability of AI performance. In addition, three examples (A8–A10) involved rule-of-thumb approaches for sample size estimation, reflecting heuristics commonly presented in applied educational sources (e.g. number of subjects per predictor or per questionnaire item). While these simplified methods are frequently used in practice, especially by non-specialists or in resource-limited settings, they may not provide optimal or fully validated estimates for all research contexts. Their inclusion nevertheless reflects real-world scenarios that researchers might encounter.

Second, we focused on the sample size estimates without verifying the appropriateness of the statistical reasoning used by the models. Moreover, because ChatGPT operates as a generative model, its process for selecting formulas and parameters is not transparent, raising concerns about the reliability of its outputs. Third, the examples were drawn from only two sources, which may introduce selection bias. Fourth, although we evaluated both ChatGPT-4.0 and ChatGPT-4o, we did not compare their performance with other AI models specifically designed for numerical tasks. Finally, there remains a possibility of data leakage, i.e. that some examples or closely related ones may have been present in ChatGPT's training data. We sought to minimize this risk by selecting examples from two distinct sources, including a published textbook, and by rephrasing the prompts. However, given the proprietary nature of the models’ training datasets, complete exclusion of prior exposure cannot be guaranteed.

## Conclusion

In this preliminary study, ChatGPT-4.0 and ChatGPT-4o provided reasonably accurate sample size estimates across a range of standard statistical scenarios. However, inconsistencies were observed, highlighting the need for cautious interpretation. These findings suggest that while AI tools may assist with basic sample size calculations, they cannot yet replace established statistical software or expert oversight. Future research should evaluate AI performance in more complex study designs, assess how different prompting strategies may affect outcomes, and explore ways to integrate AI-generated estimates into real-world research workflows as a preliminary aid rather than a definitive solution. Additionally, developing dedicated AI models with enhanced numerical reasoning capabilities may further improve accuracy in statistical applications.

## Supplementary Material

cmaf069_Supplementary_Data

## Data Availability

All data used in this study are available in Tables 1 and 2, as well as in the supplementary material.
