# Supplementary material for: ChatGPT's performance in sample size estimation: a preliminary study on the capabilities of artificial intelligence
Source: Fam Pract. 2025 Aug 26;42(5):cmaf069. doi: 10.1093/fampra/cmaf069 (PMC12411907; doi:10.1093/fampra/cmaf069)
Supplement: cmaf069_Supplementary_Data [file cmaf069_supplementary_data.pdf]

Sample Size Calculation for Examples from Verma's Book

V1. Estimating Mean Height of Male Students

What sample size is needed to estimate the mean height of male students with 95% confidence, ensuring the estimate is within 4 cm of the true mean? The population standard deviation of height (σ) is 10 cm.

V2. Estimating Mean Serum Bilirubin Levels in Infants

A researcher is interested in estimating the mean serum indirect bilirubin level in 2-week-old infants. Similar studies suggest that the standard deviation of bilirubin levels is 2.2 mg/dl. What sample size is required to estimate the mean bilirubin level within 1.2 mg/dl of the true mean, with 99% confidence?

V3. Estimating the Proportion of Non-Vegetarians

To estimate the proportion of non-vegetarians in a city, what sample size is required for a 95% confidence interval with a margin of error of 4%? In similar studies, the estimated proportion ($\hat{p}$) of non-vegetarians was 0.3.

V4. Estimating the Proportion of Non-Smokers

To develop a smoking policy, the university authority is interested in estimating the proportion of non-smokers in the university. In similar studies, the proportion of non-smokers was 0.7. What sample size is required to estimate the proportion within 3% of the true value, with 95% confidence?

V5. Testing VO_2 max Hypothesis

How large a sample is required to reject the null hypothesis (H_0: $\mu = 37$) against the alternative hypothesis (H_1: $\mu > 37$), where μ represents the population mean of VO_2 max (a measure of cardiorespiratory endurance)? The null hypothesis is tested at a 0.05 significance level, with 80% power to detect a difference of at least 1.2 units between the means ($\mu_1 - \mu_0 \geq 1.2$). The population variance, as estimated by previous studies, is 4 $ml \cdot kg^{-1} \cdot min^{-1}$.

V6. Testing Mean WBC Count Differences

In a study, the researcher aims to test whether the difference between the mean WBC counts of individuals using two different drugs is significant. The hypothesis is tested at a 0.05 significance level, with 90% power to detect a true difference as small as 40 counts between the population means. Based on earlier studies, the population variance is estimated to be 1700 counts. What sample size is required?

V7. Detecting Calcium Concentration Differences

The mean calcium concentration in a random sample of housewives aged 40–50 years is 2.5 mmol/l. What sample size is required to detect a difference between the sample mean and the population mean with 80% power at a significance level of 0.05, using a one-tailed test? The population mean and standard deviation of calcium concentration are 2.3 mmol/l and 0.5 mmol/l, respectively.

V8. Comparing Noise Levels in Two Settings

What sample size is needed to correctly reject the null hypothesis of no difference in noise levels between the Lobby and Library in a college, with 80% power? The expected mean noise levels are 62.4 dBA for the Lobby and 58.1 dBA for the Library, with an expected standard deviation (SD) of 7.8 dBA in each group. A one-tailed test will be conducted at a significance level of 0.05.

V9. Evaluating Weight Loss Program Effectiveness

An exercise scientist designs a 12-week weight loss program for housewives aged 30–40 years. How many participants are needed to detect a weight reduction of 3 kg with 80% power at a significance level of 0.05? The expected average weight of participants before starting the program is 90 kg (SD = 5 kg), and the expected average weight after 12 weeks is 87 kg (SD = 5.5 kg).

V10. Comparing Study Hours Using Mann–Whitney Test

To test whether the study hours of women are higher than those of men in a college, a researcher finds that the t-test cannot be used because the assumption of equal variance between the two groups is violated. Therefore, the researcher decides to use the Mann–Whitney test to compare the two groups. What sample size is required to detect a difference of 0.25 hours between the group means, with 90% power and $\alpha = 0.05$? The expected means and standard deviations are as follows: women (mean = 6 hours, SD = 0.5 hours) and men (mean = 5.75 hours, SD = 0.5 hours).

V11. Comparing Vaccination Proportions

Earlier studies indicate that the expected proportion of child vaccination is 0.9 in northern cities and 0.85 in southern cities. How many cases are needed to correctly reject the null hypothesis of no difference between these proportions with 80% power at a significance level of 0.05? Due to differences in population size, the sample size ratio is set as $n_2/n_1 = 1.25$, where n_1 is the sample size for northern cities and n_2 is the sample size for southern cities.

V12. Testing Correlation between Respiratory Rate and Fat%

In testing the significance of the correlation coefficient between resting respiratory rate and fat%, what sample size is required to determine whether the observed sample correlation differs from 0, with at least 45% coefficient of determination (R^2), 90% power, and a significance level of 0.05 in a two-tailed test?

V13. Testing Height-Weight Correlation in Children

The correlation between height and weight is known to be 0.55 among children aged 8–12 years. What sample size is required to correctly reject the null hypothesis that the sample correlation of 0.7 differs from 0.55, with 80% power and a significance level of 0.05 in a two-tailed test?

V14. Comparing Correlations in Men and Women

In a correlational study between IQ and Math scores, how large a sample is required to correctly reject the null hypothesis of equal correlations in men and women, given the following information?
The estimated correlation in men (ρ_1)= 0.8, the estimated correlation in women (ρ_2) = 0.68, a two-tailed test, a significance level of 0.05, a power of 0.85, and equal sample sizes in both groups.

Sample Size Calculation for Examples from Arifin's Website

A1. Estimating Mean BMI among Students

This study aims to estimate the mean BMI among students. Given that the SD of BMI is 7.5 kg/m^2, the precision is 1 kg/m^2, the confidence level is 95%, and the dropout rate is 20%, how many students should we sample?

A2. Estimating Mean Height of Male Students Estimating Prevalence of Obesity among Students

This study aims to estimate the prevalence of obesity among students. Given that the prevalence of obesity is 25% (0.25), the precision is ±5% (0.05), the confidence level is 95%, and the dropout rate is 10%, how many students should we sample?

A3. Comparing Mean BMI between Students

This study aims to compare the mean BMI between students from different years. Given that the largest SD from literature is 1.5, the expected difference is 1 unit, the significance level is 5% (0.05), the power is 80%, and the dropout rate is 30%, how many students per group should we sample?

A4. Comparing Prevalence of Obesity between Students from Different Years

This study aims to compare the prevalence of obesity between students from different years. Given that p0=35% (0.35) for the control group (1st-year students), p1=50% (0.5) for the hypothesized prevalence in final-year students, the significance level is 5% (0.05), the power is 80%, and the dropout rate is 10%, how many students per group should we sample?

A5. Comparing Mean Weight Before and After a Weight Loss Program

This study aims to compare the mean weight before and after a weight loss program (using the same individuals before and after). Given that the SD of the difference from literature is 7.5 kg, the expected difference is 5 kg (post – pre weight), the significance level is 5% (0.05), the power is 80%, and the dropout rate is 20%, how many participants should we sample?

A6. Comparing Percentage of Vaccine Uptake Before and After a Vaccine Awareness Campaign

This study aims to compare the percentage of vaccine uptake before and after a vaccine awareness campaign (using the same individuals before and after). Given that p=50% (0.5) before the campaign, p=80% (0.8) after the campaign, the significance level is 5% (0.05), the power is 80%, and the dropout rate is 10%, how many participants should we sample?

A7. Determining Pearson's Correlation between Age and Cholesterol Level

This study aims to determine the Pearson's correlation between age and cholesterol level among participants. Given that the expected correlation r is 0.55 (based on prior research), the significance level is 5% (0.05), the power is 80%, and the dropout rate is 30%, how many participants should we sample?

A8. Identifying Factors Associated with Cholesterol Levels

This study aims to identify the factors associated with cholesterol levels among participants. Using the rule-of-thumb method, the sample size is calculated as 10 subjects per independent variable: n=k×10. The study includes one numerical outcome (cholesterol) and six independent variables: three numerical (age in years, BMI in kg/m^2, and weekly physical activity in hours) and four categorical (male: yes/no, smoking: yes/no, race: Malaysian/Chinese/Indian, where race is represented by two dummy variables). Considering a 20% dropout rate, how many participants should be sampled?

A9. Identifying Factors Associated with Hypertension in Employees

This study aims to determine the factors associated with hypertension (HPT) among employees in the ABC office. Using the rule-of-thumb method, the sample size is calculated as 10 events per parameter: n=n1/p, where n1 represents the number of events (individuals with HPT) and p is the prevalence of HPT. The study includes one binary outcome (HPT) and six independent variables: three numerical (age in years, BMI in kg/m^2, weekly physical activity in hours) and four categorical (male: yes/no, smoking: yes/no, race: Malaysian/Chinese/Indian, where race is represented by two dummy variables). Given that the parameters include the independent variables, the intercept, and any interaction terms, the model contains one additional parameter (the intercept), resulting in a total of eight parameters. With a 30% dropout rate, how many participants should be sampled?

A10. Exploring the Internal Structure Validity of ABC-Q

This study aims to explore the internal structure validity of the ABC-Q questionnaire. Using the rule-of-thumb method, the sample size is calculated as five respondents per item: $n = \text{number of items} \times 5$. The questionnaire consists of 40 items. With a 30% dropout rate, how many participants should be sampled?
